# CD86 Expression by Monocytes Influences an Immunomodulatory Profile in Asymptomatic Patients with Chronic Chagas Disease

**DOI:** 10.3389/fimmu.2018.00454

**Published:** 2018-03-12

**Authors:** Bruna F. Pinto, Nayara I. Medeiros, Andrea Teixeira-Carvalho, Silvana M. Eloi-Santos, Tereza C. M. Fontes-Cal, Débora A. Rocha, Walderez O. Dutra, Rodrigo Correa-Oliveira, Juliana A. S. Gomes

**Affiliations:** ^1^Laboratório de Biologia das Interações Celulares, Departamento de Morfologia, Instituto de Ciências Biológicas, Universidade Federal de Minas Gerais, Belo Horizonte, Brazil; ^2^Instituto René Rachou, Fundação Oswaldo Cruz - FIOCRUZ, Belo Horizonte, Brazil; ^3^Instituto Nacional de Ciência e Tecnologia em Doenças Tropicais, INCT-DT, Salvador, Brazil; ^4^NUPEB, Universidade Federal de Ouro Preto, Ouro Preto, Brazil

**Keywords:** chagas disease, monocytes, immunoregulation, innate immunity, immune response

## Abstract

In the chronic phase of Chagas disease, 60% of the patients develop the asymptomatic form known as indeterminate (IND). The remaining 30% of the patients develop a life-threatening form in which digestive and/or cardiac (CARD) alterations take place. The mechanisms underlying the development of severe forms of Chagas disease remain poorly understood. It is well known that interactions between immune cells such as monocytes and lymphocytes drive immune responses. Further, the co-stimulatory molecules CD80 and CD86 expressed by monocytes and subsets induce lymphocyte activation, thereby triggering cellular immune response. Here, we revealed, for the first time, the functional-phenotypic profile of monocytes subsets in Chagas disease. Using flow cytometry, we evaluated the effect of *in vitro* stimulation with *Trypanosoma cruzi* antigens on the expression of the co-stimulatory molecules CD80 and CD86 in different monocyte subsets of patients with IND and CARD clinical forms of Chagas disease. We also assessed the expression of toll-like receptor (TLR)-2, TLR-4, TLR-9, HLA-DR, IL-10, and IL-12 in the monocyte subsets and of CTLA-4 and CD28, ligands of CD80 and CD86, in T lymphocytes. CD86 expression in all monocyte subsets was higher in IND patients when compared with non-infected (NI) individuals. After stimulation with *T. cruzi*, these patients also showed a higher frequency of CD4^+^CTLA-4^+^ T lymphocytes than NI individuals. We found an association between CD80 and CD28, and between CD86 and CTLA-4 expression, with a high frequency of regulatory T (Treg) cells in IND patients. We proposed that CD86 may be involved in immunoregulation by its association with CTLA-4 in asymptomatic patients. CD86 and CTLA-4 interaction may influence Treg activation, and this could represent a new strategy to control inflammation and tissue damage.

## Introduction

Infection with the etiologic agent of Chagas disease, the protozoan *Trypanosoma cruzi*, leads to the accumulation of parasites in the blood stream and several tissues, thus characterizing the acute phase of the disease (1–4). Afterward, the host’s immune system attempts to control the parasite replication but, as it cannot completely do so, the result is two chronic clinical outcomes (5–7). The main outcome, affecting around 60% of the patients is a life-long asymptomatic clinical form known as indeterminate (IND) (8–11). The remaining 30% of the patients, many of whom were once asymptomatic, develop severe, life-threatening forms of the disease, in which digestive and/or cardiac (CARD) alterations take place (9, 12, 13). The CARD clinical form is particularly important as it represents the clinical form associated with greater morbidity and mortality.

The mechanisms involved in the development of severe forms of Chagas disease are still poorly understood. Research conducted by our team and other laboratories revealed that important factors in the disease progression include host genetics, parasite characteristics, and host immune response (3, 12, 14, 15).

Monocytes/macrophages are innate immune cells that recognize *T. cruzi* antigens and activate lymphocytes and the immune response system (7, 16). Monocyte subsets display distinct functions and, despite their importance in regulating the immune response (17), their role in Chagas disease has not yet been determined. Moreover, the studies published so far have focused on the specific roles of monocytes and lymphocytes on the immune response induced by *T. cruzi* and did not explore the relationship between them in the development of the clinical forms of Chagas disease.

The co-stimulatory molecules CD80 and CD86 expressed on monocytes are essential for activating lymphocytes and thus adaptive immunity (18, 19). These cells constitute a heterogeneous population presenting distinct subsets according to the expression of the surface molecules CD14 and CD16 (20–22). Classical monocytes (CD14^++^CD16^−^) show high-phagocytosis capacity, have a pro-inflammatory profile and, under normal conditions, correspond to approximately 85–90% of the monocytes (22–25). Non-classical monocytes (CD14^+^CD16^++^) show an anti-inflammatory profile and a patrol behavior *in vivo* (22, 25). Molecular studies showed that intermediate monocytes (CD14^++^CD16^+^) are between the classical and non-classical monocyte subsets and express the highest levels of the major histocompatibility complex class II (26). However, there are no studies regarding the functional-phenotypic profile of monocyte subsets in Chagas disease.

The two main receptors present on the surface of T lymphocytes that bind CD80 and CD86 are CD28 and cytotoxic T-lymphocyte associated protein 4 (CTLA-4). Binding to CD28 activates lymphocytes and consequently enhances the immune response, whereas binding to CTLA-4 inhibits activation of lymphocytes, thereby downregulating immunity (19, 27–29). T lymphocytes play a crucial role in the establishment and development of human Chagas disease, displaying both immunoregulatory and effector functions. These functions determine the disease dynamics and are involved in the polarity found in the clinical forms of the disease (30–34).

Previous studies evaluated the expression of CD80 and CD86 co-stimulatory molecules by monocytes of Chagas patients (35). However, no study to date explored the effect of the expression of these molecules by classical (CD14^++^CD16^−^), intermediate (CD14^++^CD16^+^), and non-classical (CD14^+^CD16^++^) monocyte subsets on the activation of T helper (Th) type 1 (Th1), type 2 (Th2), type 17 (Th17), and regulatory T (Treg) cells. We addressed this knowledge gap by profiling the expression of CD80 and CD86 by monocyte subsets of IND and CARD individuals in the activation of Th cells following the challenge by *T. cruzi* antigens. Ultimately, we aimed to characterize the functional-phenotypic profile of monocyte subsets and understand the CD80/CD86 co-stimulatory expression by monocyte subsets in the protective and/or regulatory immunity against *T. cruzi* infection to further understand Chagas disease progression.

## Materials and Methods

### Study Population

Nineteen patients who agreed to participate in this study were selected at René Rachou Institute ambulatory, Oswaldo Cruz Foundation (FIOCRUZ), Belo Horizonte, Minas Gerais, Brazil. Patients were considered infected when they tested positive for Chagas disease by at least two different serological tests (indirect immunofluorescence, ELISA, or indirect hemagglutination).

The patients infected with *T. cruzi* were grouped as IND and CARD. The IND (*n* = 10) group included individuals tested positive for Chagas disease albeit with no significant alterations in electrocardiography, chest X-ray, echocardiogram, esophagogram, and barium enema. The CARD (*n* = 9) group presented dilated cardiomyopathy, characterized by the echocardiographic finding of a dilated left ventricle with impaired ventricular systolic function, which were classified as belonging to the group CARD V, as previously reported (36). Left ventricular end-diastolic diameter/body surface area ≥31 mm (64.8 ± 5.9 mm) and left ventricular ejection fraction <55% (34 ± 10%) were used as echocardiographic parameters of Chagas dilated cardiomyopathy. Normal healthy individuals that tested negative for the infection were included as a control group [non-infected (NI), *n* = 6]. All patients included in this study were between 30 and 75 years of age.

### Ethics Statement

This study was approved by the Ethics Committee of the René Rachou Institute, FIOCRUZ (15/2011). All enrolled patients signed an informed consent form prior to the inclusion in the study.

### *T. cruzi* Soluble Antigen Preparations (TRIPO)

Trypomastigote forms were obtained from culture of LLC cells maintained in RPMI-1640 medium (Gibco, Thermo Fisher Scientific, USA) supplemented with 10% fetal bovine serum, as previously described (33). Parasites were subjected to rupture and homogenization in cold phosphate-buffered saline (PBS, Sigma, USA), using a glass homogenizer and Teflon pestle, on ice to prevent overheating. Subsequently, the suspensions were centrifuged at 23.000 *g* for 60 min at 4°C. The supernatant was collected, dialyzed for 24 h at 4°C against PBS, and sterilized by filtration on 0.2 μm-pore-size membranes. The protein concentration was measured by Nanodrop (Thermo Scientific, USA) and the material was separated into aliquots and stored at −70°C until use.

### Whole Blood Cultures

Whole blood collected in sodic heparin was cultured for 18 h at 37°C and 5% CO_2_ with medium alone (RPMI-1640 supplemented with 1.6% l-glutamine, 3% antibiotic–antimycotic, 5% of AB Rh-positive heat-inactivated normal human serum) or medium plus 20 µL/mL of soluble *T. cruzi* antigens (TRIPO). Previously, 20 µL of Brefeldin A (Sigma, USA) at 1 mg/mL concentration was added to the tubes and they were incubated for another 4 h at 37°C and 5% CO_2_. Subsequently, the culture tubes were incubated for 15 min after addition of 200 µL of EDTA (Sigma, USA) to reach the concentration of 2 mM. Then, 3 mL of PBS-W (PBS pH 7.4, containing 0.5% BSA, and 0.1% sodium azide) were added to the tubes and the samples were centrifuged at 400 *g* for 10 min at 18°C. The supernatant was aspirated leaving a final volume of 2.5 mL. Subsequently, 200 µL of this blood was added in polystyrene tubes (Falcon, USA) with corresponding surface antibodies and was incubated for 30 min. After the incubation, the samples were lysed and fixed in 2 mL of FACS Lysing Solution (BD, USA) for 10 min at room temperature. The samples were washed in PBS-W and subsequently, 2.5 mL of PBS-P (PBS, pH 7.4 containing 0.5% BSA, 0.1% sodium azide, and 0.5% saponin) were added followed by an incubation of 30 min. To detect intracellular markers, 2 µL of anti-cytokine antibody was added to each tube and incubated for 1 h at room temperature. Then, the cells were washed with 1 mL PBS-W and centrifuged at 400 *g* for 10 min at 18°C. At the end, 200 µL of fixative solution (Sigma, USA) were added in the tubes.

Samples containing the cell suspension were used to acquire data on a flow cytometer (FACS LSR Fortessa, BD, USA). A total of 60,000 events inside the lymphocyte population were analyzed using size (FSC) and granularity (SSC) parameters. Antibodies were conjugated to FITC, PERCP (or PE-Cy5 or PERCP-Cy5.5), antigen-presenting cell (APC), APCCy7, PE-Cy7, and BV421. The analyzed molecules were: CD4 (RPA-T4), CD28 (CD28.2), CTLA-4 (BNI3), CD14 (MφP9), CD16 (3G8), CD80 (2D10), CD86 (2331), Tbet (4B10), GATA-3 (L50-823), RORγT (Q21-559), CD25 (M-A251), FOXP3 (PCH101), IFN-γ (25723.11), IL-4 (8D4-8), IL-10 (JES3-19F1), IL-17 (eBio64DEC17), toll-like receptor (TLR)-2 (TL2.1), TLR-4 (HTA125), TLR-9 (eB72-1665), HLA-DR (G46-6), and IL-12 (C11.5).

### Statistical Analysis

To verify statistical differences between control and TRIPO cultures, a paired analysis was performed employing the Wilcoxon test, followed by Rank through the statistical program GraphPad Prism version 5.0 software (San Diego, CA, USA). Differences between the clinical forms in the control and TRIPO cultures were determined with the Kruskal–Wallis test.

The association between the monocytes and lymphocytes subsets was determined from the linear regression, considering the coefficient of determination (*R*^2^) for the quality of fit and the *F* test to measure the variance between pairs (*p* < 0.05). Correlation analysis was done using the Spearman’s (ρ) coefficient contained in the JMP software version 5.0. The defined confidence interval was 95% and significant statistical differences were considered when *p* < 0.05.

## Results

### Chagas Patients Show Different Frequencies of Monocyte Subsets in Control and *T. cruzi*-Stimulated Cultures

**Figure 1:**
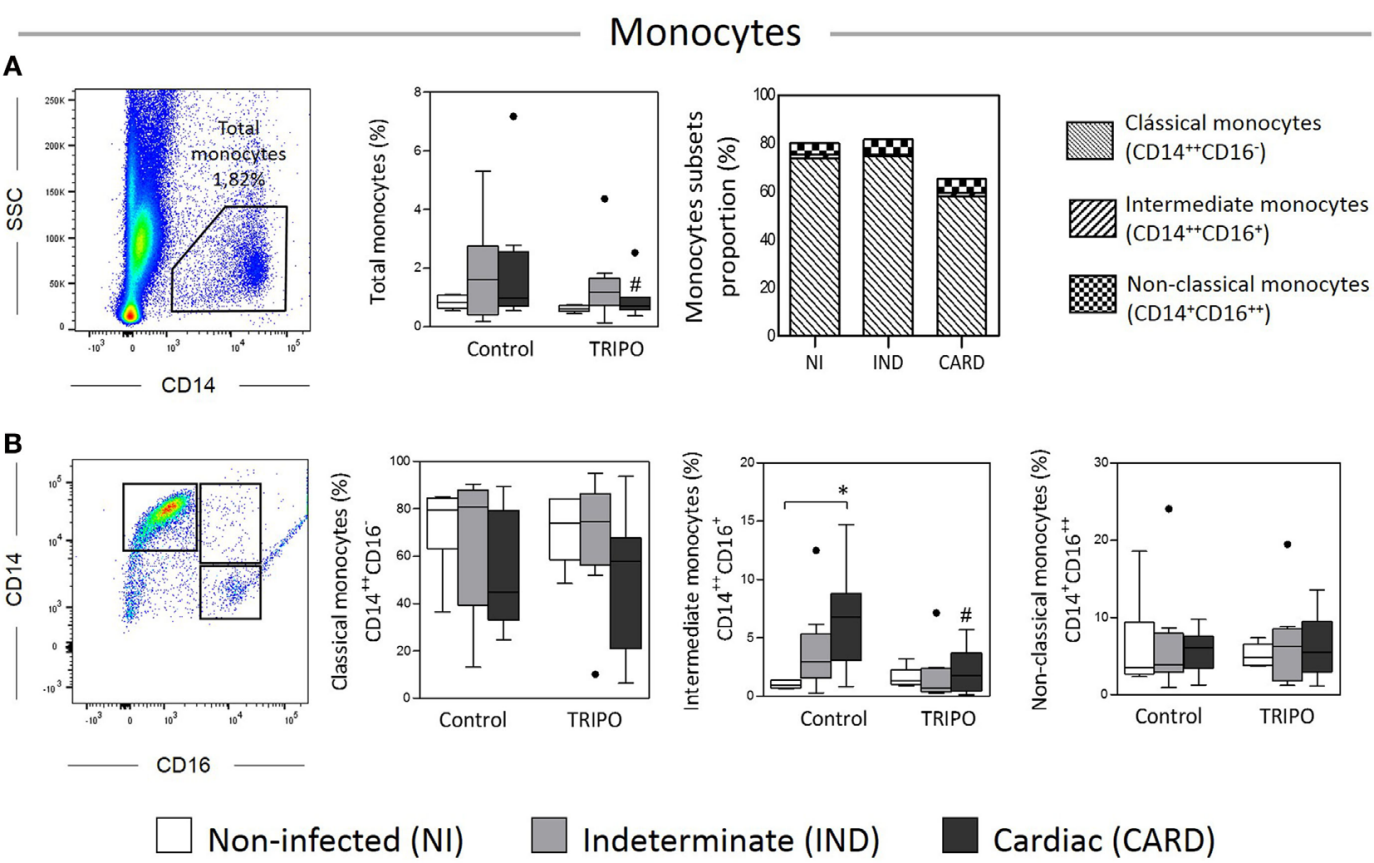
Analysis of total monocytes and subsets. Flow cytometry gate strategy of non-infected (NI) individuals and frequency of total monocytes **(A)**, classical (CD14^++^CD16^−^), intermediate (CD14^++^CD16^+^), and non-classical (CD14^+^CD16^++^) subsets in culture without stimulation (control) and after *in vitro* stimulation with *T. cruzi* antigens (TRIPO) **(B)**. The groups evaluated were NI individuals (*n* = 6), indeterminate (IND, *n* = 10), and cardiac (CARD, *n* = 9) clinical forms of Chagas disease. Significant differences (*p* < 0.05) between groups are evidenced by lines and asterisks (*) according Kruskal–Wallis test, followed by Dunn’s *post hoc* test. The # sign indicates significant difference between control and TRIPO cultures using Wilcoxon signed-rank test. Boxes show the median and interquartile ranges, whiskers indicate the highest and lowest observation, and dots represent the outliers.

The frequency of total monocytes and monocyte subsets obtained from Chagas disease patients and healthy individuals is presented in Figures 1A,B. Our results showed a significant reduction in the frequency of total monocytes in the CARD group after *in vitro* stimulation with *T. cruzi* antigens (TRIPO culture) when compared with the control culture. We observed a higher frequency of intermediate monocytes in the CARD group when compared with the NI group in control culture. Interestingly, there was a reduction in the frequency of this monocyte subset in the CARD group challenged with *T. cruzi* in comparison with CARD group cultured in control medium. Other significant differences were not observed.

When we analyzed the proportions of the monocyte subsets of Chagas disease patients and healthy individuals in TRIPO cultures, we found that the NI, IND, and CARD groups presented a higher proportion of classical than intermediate and non-classical monocytes. We also observed a small increase in the proportion of non-classical monocytes in the IND group when compared with the individuals of the NI and CARD groups. A higher proportion of intermediate monocytes were found in the CARD group in comparison with NI and IND groups.

### Lower Expression of TLR in the Monocytes of Chagas Disease Patients

Toll-like receptors are crucial to recognize structures conserved in microorganisms such as PAMPs (37). In Chagas disease, TLR-2 is associated with the initiation of the immune response by recognizing GPI anchor-linked mucin molecules (38), TLR-4 with the recognition of glycoinositolphospholipid in the parasite cell surface (39), and TLR-9 receptors with the identification of unmethylated CpG motifs in the *T. cruzi* genome (38, 40). Thus, TLRs are of great importance for parasite recognition and to drive the production of cytokines in response to the microorganism (41).

**Figure 2:**
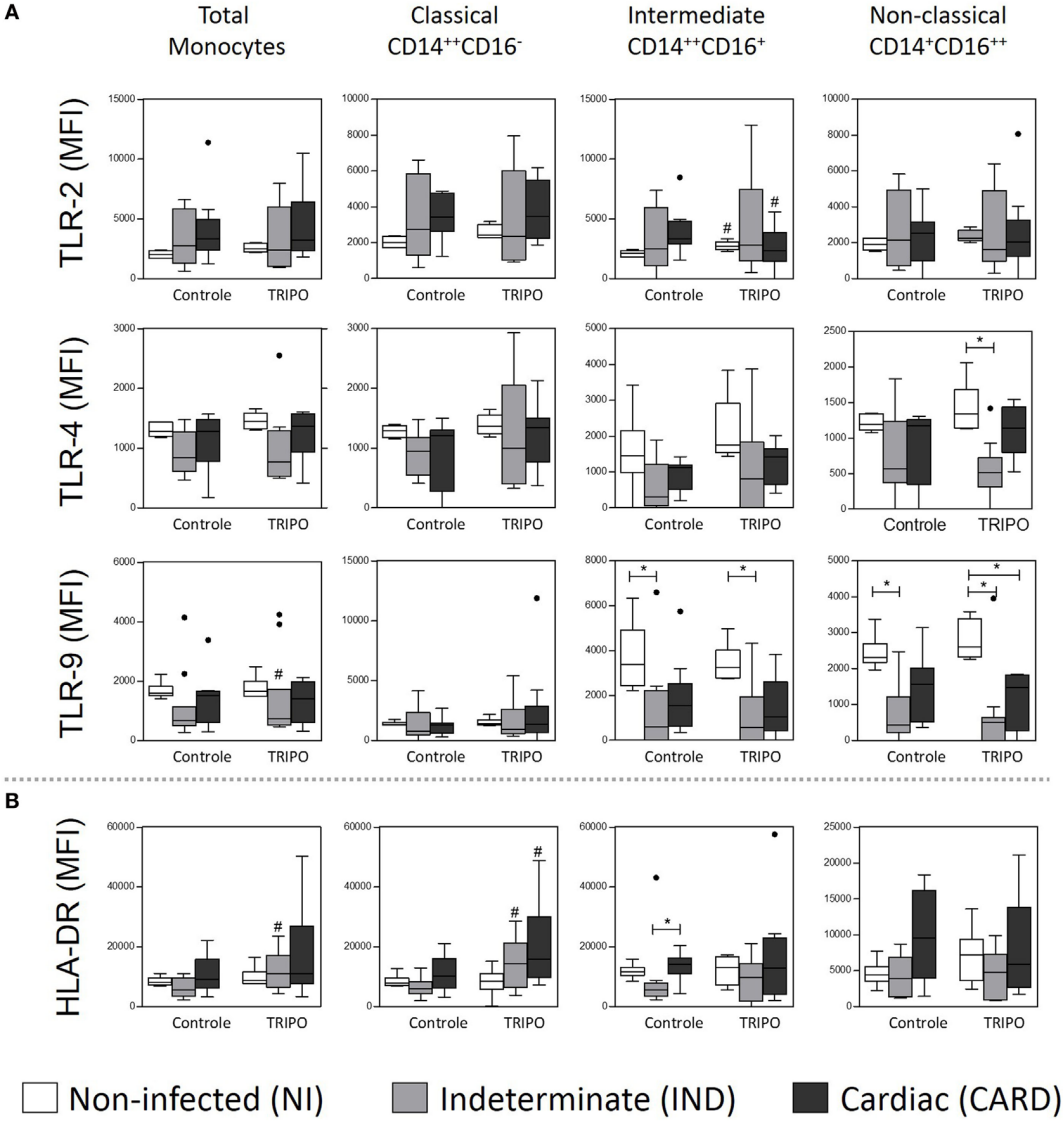
Expression of the recognition and activation molecules of total monocytes and subsets. Expression of toll-like receptors (TLR) **(A)**, and HLA-DR **(B)** by total monocytes, classical (CD14^++^CD16), intermediate (CD14^++^CD16^+^), and non-classical (CD14^+^CD16^++^) monocytes subsets in cultures without stimulation (control) and after *in vitro* stimulation with *T. cruzi* antigens (TRIPO). The groups evaluated were non-infected (NI) individuals (*n* = 6), indeterminate (IND, *n* = 10), and cardiac (CARD, *n* = 9) clinical forms of Chagas disease. Significant differences (*p* < 0.05) between groups are evidenced by lines and asterisks (*) according Kruskal–Wallis test, followed by Dunn’s *post hoc* test. The # sign indicates significant difference between control and TRIPO cultures using Wilcoxon signed-rank test. Boxes show the median and interquartile ranges, whiskers indicate the highest and lowest observation, and dots represent the outliers. MFI, mean fluorescence intensity.

Stimulation with *T. cruzi* led to an increased expression of TLR-2 on intermediate monocytes of the NI group and a reduced expression of this receptor on the same monocyte subset of the CARD group, when compared with control cultures. Challenge with *T. cruzi* also led to lower expression of TLR-4 on non-classical monocytes of the IND patients when compared with the NI group. Additionally, after *in vitro* stimulation with *T. cruzi* antigens, total monocytes of IND individuals showed high-expression TLR-9. In contrast, we observed a lower expression of this receptor on intermediate and non-classical monocytes of the IND group when compared with NI individuals, in both control and TRIPO cultures (Figure 2A). Other significant differences were not observed.

### Monocyte Subsets of Patients with Chagas Disease Have Different HLA-DR Expression Levels

After *in vitro* stimulation with *T. cruzi* antigens, total monocytes of IND patients presented higher expression of HLA-DR when compared with the control culture. Classical monocytes of IND and CARD groups in TRIPO culture presented a higher expression of this molecule, in comparison with control cultures. The results also showed lower expression of HLA-DR on intermediate monocytes of IND patients when compared with the CARD group in control cultures (Figure 2B).

### *In Vitro* Stimulation of Monocytes Subsets with *T. cruzi* Upregulates the Expression of CD80 and CD86 in Chagasic Patients

**Figure 3:**
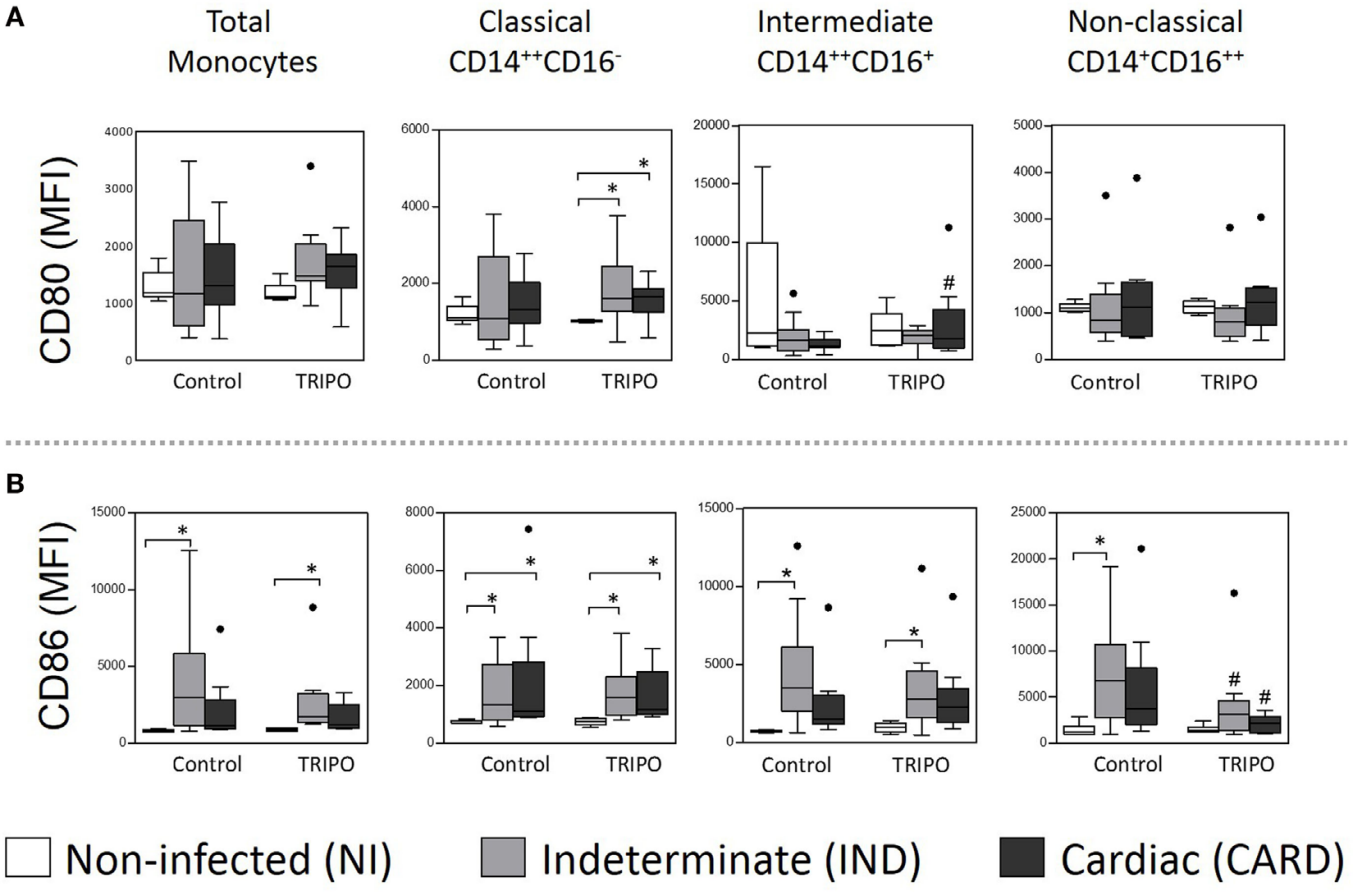
Expression of the co-stimulatory molecules of total monocytes and subsets. Expression of CD80 **(A)**, and CD86 **(B)** co-stimulatory molecules by total monocytes, classical (CD14^++^CD16), intermediate (CD14^++^CD16^+^), and non-classical (CD14^+^CD16^++^) monocytes subsets in cultures without stimulation (control) and after *in vitro* stimulation with *T. cruzi* antigens (TRIPO). The groups evaluated were non-infected (NI) individuals (*n* = 6), indeterminate (IND, *n* = 10), and cardiac (CARD, *n* = 9) clinical forms of Chagas disease. Significant differences (*p* < 0.05) between groups are evidenced by lines and asterisks (*) according Kruskal–Wallis test, followed by Dunn’s *post hoc* test. The # sign indicates significant difference between control and TRIPO cultures using Wilcoxon signed-rank test. Boxes show the median and interquartile ranges, whiskers indicate the highest and lowest observation, and dots represent the outliers. MFI, mean fluorescence intensity.

Next, we evaluated the effect of circulating monocytes of chronic Chagasic patients on the activation of T cells. To this end, we characterized the expression of the co-stimulatory molecules CD80 and CD86 on short-term *in vitro* cultures of whole blood samples in the presence of *T. cruzi* antigens (Figures 3A,B).

In TRIPO stimulated cultures, the expression of CD80 on the classical monocytes of IND and CARD groups were higher than in the NI group. This co-stimulatory molecule was also highly expressed on intermediate monocytes of the CARD group challenged with *T. cruzi* antigens, when compared with the control culture. Significant differences were not observed in the expression of CD80 on non-classical monocytes (Figure 3A). Total monocytes and all the subsets of IND patients presented higher expression of CD86 when compared with NI group in control and TRIPO cultures, except in the case of non-classical monocytes after *in vitro* stimulation with *T. cruzi* antigens. CARD patients showed high-CD86 expression only on classical monocytes when compared with NI group in both control and TRIPO cultures. We also observed a reduced expression of this molecule on non-classical monocytes of IND and CARD groups after *T. cruzi* stimulation (Figure 3B). Other significant differences were not observed.

**Figure 4:**
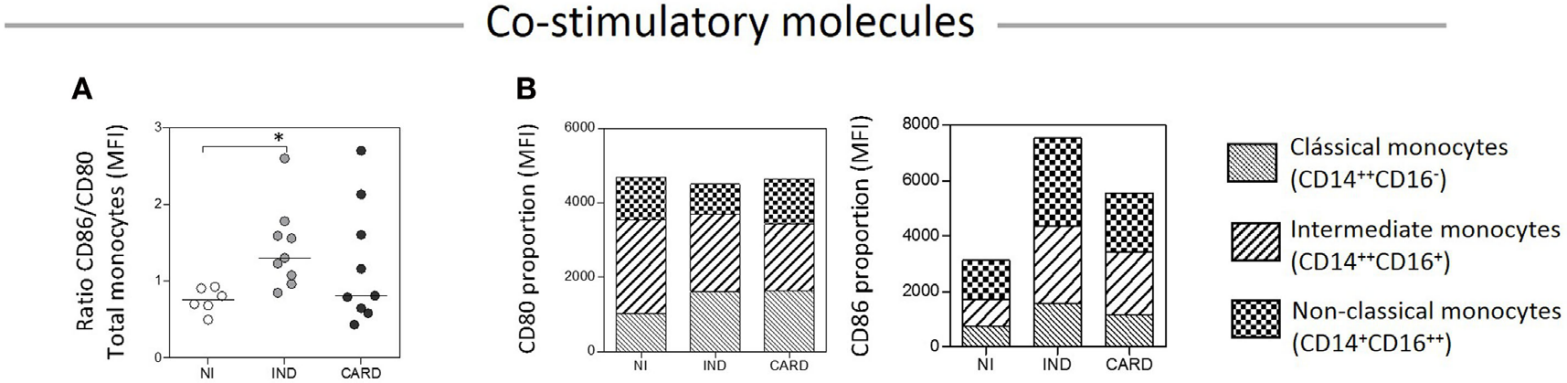
Ratio/proportion of CD80 and CD86 expression by monocytes. Ratio of CD86/CD80 expression by total monocytes **(A)**, and proportion of these co-stimulatory molecules by the monocyte subsets **(B)**. The groups evaluated were non-infected (NI) individuals (*n* = 6), indeterminate (IND, *n* = 10), and cardiac (CARD, *n* = 9) clinical forms of Chagas disease. Significant differences (*p* < 0.05) between groups are evidenced by lines and asterisks (*) according Kruskal–Wallis test, followed by Dunn’s *post hoc* test. MFI, mean fluorescence intensity.

To compare the expression of CD86 and CD80 on monocytes—total and subsets—we calculated a relative expression index, considering the CD86/CD80 ratio and the proportion through the mean intensity fluorescence of these molecules in TRIPO cultures of NI, IND, and CARD individuals (Figures 4A,B). Our results showed higher CD86/CD80 expression ratio on total monocytes of IND compared with NI group. The highest CD80 expression was observed on the intermediate monocytes of the NI group. As the disease progresses, CD80 expression decreased on the intermediate and non-classical subsets and increased on the classical subset of the IND group. Further, in the CARD group, CD80 expression shifted from intermediate to non-classical monocytes while the expression observed on the classical monocyte subset remained stable. We found a similar proportion of CD86 expression on classical, intermediate, and non-classical monocytes of the NI group. As the disease progresses, CD86 expression increased on all monocyte subsets of IND patients in comparison with those of the NI group, and decreased on the subsets of the CARD group in comparison with the IND group, albeit CD86 expression remained higher than that observed in the NI group (Figure 4).

### Asymptomatic Patients with Chagas Disease Showed a Balance between IL-12 and IL-10 Expression

In Chagas disease, while the cytokine IL-12 leads to the activation of a Th1-lymphocyte-mediated response, the cytokine IL-10 contributes to the inhibition of IL-12 production by monocytes, lymphocytes, and dendritic cells, leading to regulation of immune response (32).

**Figure 5:**
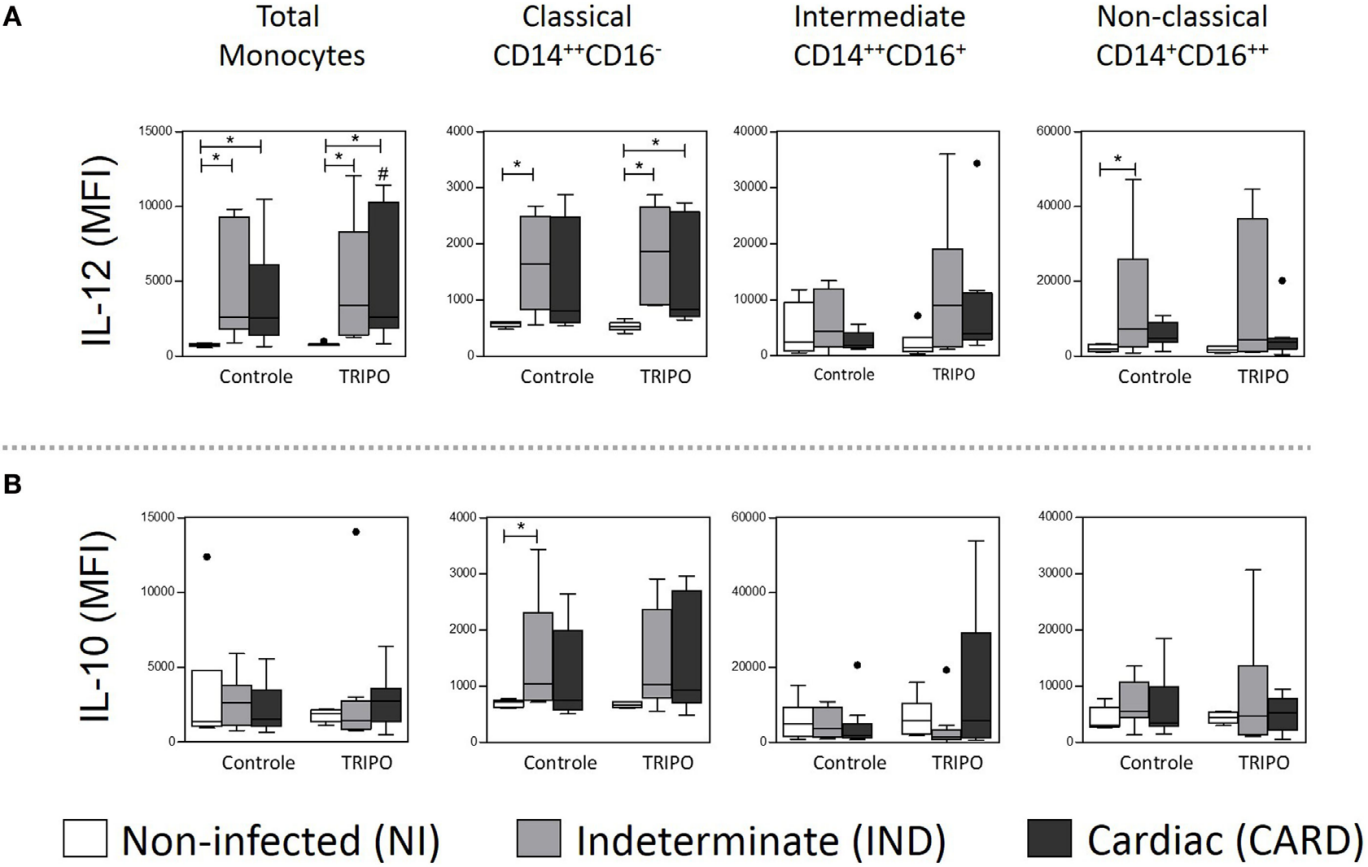
Expression of cytokines of total monocytes and subsets. Expression of IL-10 **(A)**, and IL-12 **(B)** by total monocytes, classical (CD14^++^CD16), intermediate (CD14^++^CD16^+^), and non-classical (CD14^+^CD16^++^) monocytes subsets in cultures without stimulation (control) and after *in vitro* stimulation with *T. cruzi* antigens (TRIPO). The groups evaluated were non-infected (NI) individuals (*n* = 6), indeterminate (IND, *n* = 10), and cardiac (CARD, *n* = 9) clinical forms of Chagas disease. Significant differences (*p* < 0.05) between groups are evidenced by lines and asterisks (*) according Kruskal–Wallis test, followed by Dunn’s *post hoc* test. The # sign indicates significant difference between control and TRIPO cultures using Wilcoxon signed-rank test. Boxes show the median and interquartile ranges, whiskers indicate the highest and lowest observation, and dots represent the outliers. MFI, mean fluorescence intensity.

We found that the total monocytes of patients with Chagas disease present a higher expression of IL-12 when compared with the NI group, in both control and TRIPO cultures. After *in vitro* stimulation with *T. cruzi* antigens, total monocytes of the CARD group showed a lower expression of this cytokine (Figure 5A).

When we analyzed the monocyte subsets, we observed a higher expression of IL-12 on classical monocytes of IND patients when compared with the NI group in control culture and, after *in vitro* stimulation with *T. cruzi* antigens, in IND and CARD patients in comparison with the NI group. We also detected higher expression of IL-12 on non-classical monocytes of IND patients when compared with the NI group in control culture (Figure 5A). In addition, classical monocytes showed higher expression of IL-10 only in IND patients, when compared with NI group in control culture (Figure 5B). Other significant differences were not observed.

### CTLA-4 Molecule Is Upregulated in CD4^+^ T Lymphocytes in the IND Clinical Form of Chagas Disease

**Figure 6:**
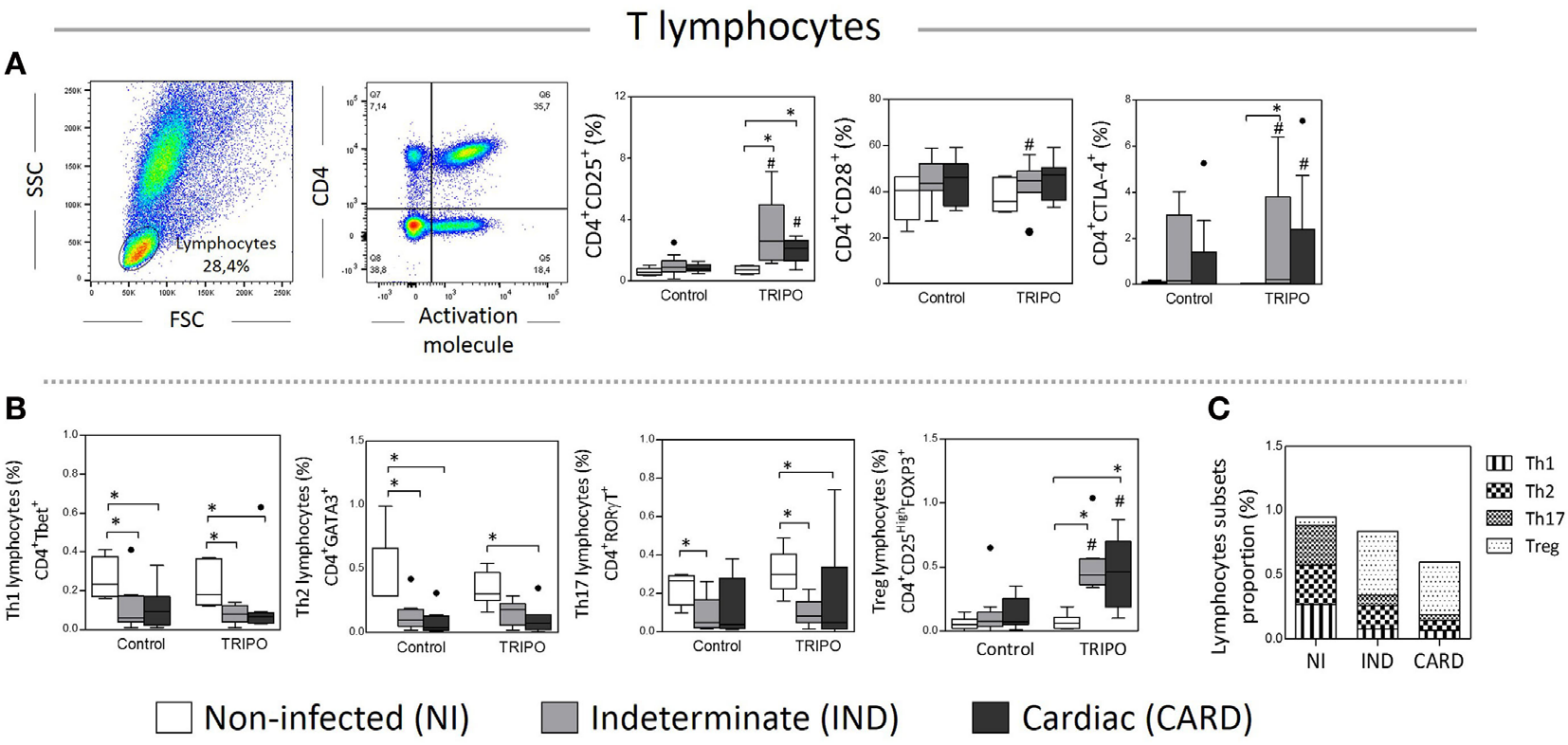
Analysis of lymphocytes and subsets. Flow cytometry gate strategy of non-infected (NI) individuals is represented. Frequency of CD4^+^ lymphocytes expressing CD25, CD28, and CTLA-4 **(A)** in culture without stimulation (control) and after *in vitro* stimulation with *T. cruzi* antigens (TRIPO). Frequency of Th1, Th2, Th17, and T regulatory (Treg) lymphocytes subsets **(B)** and, proportion of lymphocyte subsets **(C)**. The groups evaluated were NI individuals (*n* = 6), indeterminate (IND, *n* = 10), and cardiac (CARD, *n* = 9) clinical forms of Chagas disease. Significant differences (*p* < 0.05) between groups are evidenced by lines and asterisks (*) according Kruskal–Wallis test, followed by Dunn’s *post hoc* test. The # sign indicates the significant difference between control and TRIPO cultures using Wilcoxon signed-rank test. Boxes show the median and interquartile ranges, whiskers indicate the highest and lowest observation, and dots represent the outliers.

CD25, CD28, and CTLA-4 are important activation molecules present on the surface of T lymphocytes that are essential to the adaptive immune response (19, 27, 42). We evaluated the expression of these molecules in CD4^+^ T cells of NI, IND, and CARD individuals. We found a higher frequency of CD4^+^ CD25^+^ T lymphocytes in TRIPO cultures of IND and CARD patients in comparison with NI group and in comparison with the frequency observed in the control cultures. We also detected a slight but significant increase of CD4^+^CD28^+^ T lymphocytes frequency in the TRIPO culture compared with control cultures of IND patients. When we evaluated the frequency of CD4^+^ CTLA-4^+^ T lymphocytes, we observed that, after stimulation with *T. cruzi* antigens, IND and, CARD patients showed an increased frequency of these cells in comparison with control culture. Interestingly only IND patients showed a higher frequency of CD4^+^ CTLA-4^+^ T lymphocytes when compared with NI group in TRIPO cultures (Figure 6A). Other significant differences were not observed.

### Chagasic Patients Decrease Th1, Th2, and Th17 and Increase of Treg Cells after Stimulation with *T. cruzi* Antigens

The immune response mediated by CD4^+^ T lymphocytes is highly important for the development of Chagas disease involving the Th1, Th2, Th17, or Treg CD4^+^ lymphocytes that differ in their functions, secreted cytokine pattern, and specific expression of transcription factors (16, 43). Therefore, we evaluated the frequency of Th1, Th2, Th17, and Treg lymphocytes in the peripheral blood following *in vitro* stimulation with *T. cruzi* antigens (Figure 6B). The data show a general decrease in the frequency of Th1, Th2, and Th17 lymphocytes of Chagasic patients in comparison with NI individuals, in the control, and TRIPO cultures. In contrast, the frequency of Treg lymphocytes was significantly higher in IND and CARD groups compared with NI individuals after *in vitro* stimulation with *T. cruzi* antigens and in comparison, to the frequency observed in the control cultures (Figure 6B). Other significant differences were not observed.

When we analyzed the proportions of lymphocyte subsets in Chagas disease patients and healthy individuals in TRIPO cultures, we found that NI group presented similar proportions of Th1, Th2, and Th17 lymphocytes and a small proportion of Treg cells. As the disease progresses, the proportion of Th1, Th2, and Th17 cells diminishes in IND and CARD individuals. In contrast, the proportion of Treg cells increases dramatically in Chagasic patients compared with NI group, mainly in IND group (Figure 6C).

**Figure 7:**
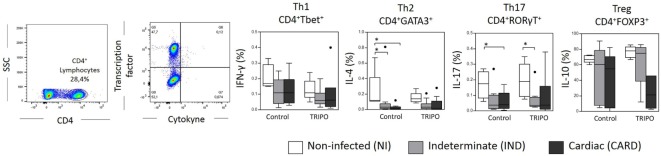
Frequency of cytokines^+^ lymphocytes subsets. Flow cytometry gate strategy of non-infected (NI) individuals is represented. Expression of IFN-γ, IL-4, IL-17, and IL-10 by CD4^+^ T lymphocytes that characterize Th1, Th2, Th17, and regulatory T subsets, respectively. The groups evaluated were NI individuals (*n* = 6), indeterminate (IND, *n* = 10), and cardiac (CARD, *n* = 9) clinical forms of Chagas disease. Significant differences (*p* < 0.05) between groups are evidenced by lines and asterisks (*) according Kruskal–Wallis test, followed by Dunn’s *post hoc* test. Boxes show the median and interquartile ranges, whiskers indicate the highest and lowest observation, and dots represent the outliers.

The expression of IFN-γ, IL-4, IL-17, and IL-10 by CD4^+^ T lymphocytes is a key factor for the differentiation and characterization of Th1, Th2, Th17, and Treg subsets, respectively (16). We found a lower frequency of Th2 IL-4^+^ lymphocytes in control cultures of IND and CARD patients in comparison with NI group. We also detected a lower frequency of Th17 IL-17^+^ lymphocytes in control and TRIPO cultures of CARD and IND individuals when compared with NI group, respectively. No significant differences were observed regarding Th1 IFN-γ^+^ lymphocytes and Treg IL-10^+^ lymphocytes (Figure 7).

### CD86 Co-Stimulatory Molecule May Induce an Immunomodulatory Treg Response in IND Patients

As detailed above, patients with the IND clinical form of Chagas disease show a higher expression of CD86 than CD80 co-stimulatory molecules by all monocyte subsets. The interaction between CD80 or CD86 with CD28 activates lymphocytes and enhances the immune response, whereas their binding to CTLA-4 inhibits the activation of lymphocytes activation thus downmodulating the immune response (35). We investigated this interaction by performing regression and correlation analyzes.

First, we evaluated the interaction between CD80/CD86 of total monocytes and CD28/CTLA-4 in TRIPO cultures of NI, IND, and CARD individuals and found a significant association between CD80^+^ and CD4^+^ CD28^+^ T cells of the NI group (*R*^2^ = 0.73/*p* = 0.03). We also detected, in these individuals, a significant positive correlation between CD80^+^ and CTLA-4^+^ T cells (ρ = 0.93/*p* = 0.008). A significant association between CD86^+^ and CTLA-4^+^ T cells was found in CARD patients (*R*^2^ = 0.81/*p* = 0.001) (Table 1).

**Table 1:** Linear regression and correlation analysis of CD80 and CD86 co-stimulatory molecules by monocytes and their subsets with CD28 and CTLA-4 molecules and Th1 Th2, Th17, and regulatory T (Treg) cells.

			Total monocytes		Classical monocytes		Intermediate monocytes		Non-classical monocytes	

			*R*^2^ (*p*-value)	ρ (*p*-value)	*R*^2^ (*p*-value)	ρ (*p*-value)	*R*^2^ (*p*-value)	ρ (*p*-value)	*R*^2^ (*p*-value)	ρ (*p*-value)
Non-infected	CD80									
		CD28	0.73 (0.03)*	−0.03 (0.96)	0.20 (0.38)	−0.09 (0.87)	0.84 (0.01)*	−0.37 (0.47)	0.001 (0.94)	0.23 (0.66)
		CTLA-4	0.58 (0.08)	0.93 (0.008)*	0.002 (0.93)	0.20 (0.70)	0.28 (0.28)	0.43 (0.39)	0.16 (0.43)	0.03 (0.96)
		Th1	0.07 (0.62)	0.086 (0.87)	0.21 (0.34)	−0.43 (0.40)	0.04 (0.70)	0.26 (0.63)	0.18 (0.40)	0.37 (0.47)
		Th2	0.05 (0.68)	−0.54 (0.27)	0.19 (0.38)	−0.43 (0.40)	0.01 (0.88)	0.09 (0.87)	0.03 (0.76)	0.029 (0.96)
		Th17	0.04 (0.70)	0.029 (0.96)	0.36 (0.21)	−0.77 (0.07)	0.03 (0.75)	0.09 (0.87)	0.13 (0.48)	0.26 (0.63)
		Treg	0.33 (0.23)	−0.94 (0.00)*	0.27 (0.29)	0.2 (0.70)	0.20 (0.38)	− 0.31 (0.54)	0.01 (0.89)	− 0.06 (0.87)
	CD86									
		CD28	0.01 (0.84)	0.03 (0.96)	0.22 (0.34)	−0.64 (0.17)	0.38 (0.19)	−0.54 (0.27)	0.005 (0.89)	−0.26 (0.62)
		CTLA−4	0.06 (0.65)	−0.26 (0.62)	0.48 (0.13)	−0.14 (0.79)	0.04 (0.71)	−0.23 (0.66)	0.03 (0.76)	−0.20 (0.70)
		Th1	0.27 (0.29)	0.43 (0.40)	0.02 (0.78)	−0.2 (0.7)	0.02 (0.80)	0.14 (0.79)	0.60 (0.07)	0.49 (0.33)
		Th2	0.45 (0.14)	0.43 (0.40)	0.41 (0.17)	0.54 (0.27)	0.04 (0.71)	0.43 (0.40)	0.56 (0.09)	0.54 (0.27)
		Th17	0.04 (0.71)	0.086 (0.87)	0.048 (0.68)	−0.25 (0.62)	0.01 (0.86)	0.31 (0.54)	0.64 (0.06)	−0.14 (0.79)
		Treg	0.22 (0.35)	0.66 (0.16)	0.43 (0.16)	0.77 (0.07)	0.01 (0.86)	−0.08 (0.87)	0.79 (0.02)*	0.6 (0.21)

Indeterminate	CD80									
		CD28	0.24 (0.18)	−0.58 (0.10)	0.20 (0.23)	−0.57 (0.11)	0.009 (0.78)	−0.18 (0.64)	0.004 (0.87)	0.07 (0.86)
		CTLA−4	0.13 (0.39)	0.40 (0.32)	0.13 (0.38)	0.29 (0.49)	0.57 (0.03)*	0.86 (0.007)*	0.19 (0.29)	−0.31 (0.46)
		Th1	0.10 (0.40)	0.23 (0.56)	0.03 (0.66)	0.06 (0.88)	0.01 (0.91)	0.17 (0.67)	0.08 (0.45)	0.19 (0.62)
		Th2	0.28 (0.14)	0.47 (0.2)	0.41 (0.06)	0.75 (0.02)*	0.19 (0.23)	0.46 (0.22)	0.27 (0.15)	−0.59 (0.09)
		Th17	0.31 (0.12)	0.39 (0.29)	0.26 (0.16)	0.40 (0.28)	0.23 (0.18)	0.40 (0.28)	0.06 (0.54)	−0.06 (0.88)
		Treg	0.10 (0.42)	−0.73 (0.02)*	0.07 (0.49)	−0.63 (0.07)	0.00 (0.98)	−0.38 (0.30)	0.02 (0.72)	0.59 (0.09)
	CD86									
		CD28	0.16 (0.29)	−0.42 (0.26)	0.003 (0.88)	−0.08 (0.83)	0.07 (0.49)	−0.23 (0.55)	0.10 (0.41)	−0.32 (0.41)
		CTLA−4	0.17 (0.30)	0.60 (0.12)	0.55 (0.04)*	0.67 (0.07)	0.10 (0.45)	0.29 (0.49)	0.10 (0.44)	0.45 (0.26)
		Th1	0.17 (0.26)	0.13 (0.75)	0.004 (0.87)	0.17 (0.67)	0.04 (0.60)	−0.08 (0.83)	0.15 (0.31)	0.18 (0.64)
		Th2	0.27 (0.26)	0.54 (0.13)	0.08 (0.46)	0.24 (0.54)	0.26 (0.16)	0.41 (0.28)	0.33 (0.10)	0.68 (0.04)*
		Th17	0.30 (0.13)	0.29 (0.44)	0.03 (0.67)	0.21 (0.59)	0.38 (0.08)	0.49 (0.18)	0.29 (0.13)	0.24 (0.53)
		Treg	0.11 (0.38)	0.66 (0.05)	0.22 (0.20)	−0.78 (0.01)*	0.12 (0.36)	−0.53 (0.14)	0.10 (0.40)	−0.73 (0.03)*

Cardiac	CD80									
		CD28	0.003 (0.89)	0.06 (0.86)	0.003 (0.89)	0.07 (0.87)	0.16 (0.29)	0.60 (0.09)	<0.01 (0.10)	−0.55 (0.13)
		CTLA-4	0.05 (0.55)	−0.09 (0.81)	0.05 (0.55)	−0.09 (0.81)	0.10 (0.41)	−0.44 (0.24)	0.38 (0.08)	−0.80 (0.00)*
		Th1	0.02 (0.69)	−0.37 (0.33)	0.02 (0.68)	−0.37 (0.33)	0.03 (0.64)	−0.35 (0.36)	0.004 (0.86)	−0.43 (0.24)
		Th2	0.04 (0.59)	0.20 (0.60)	0.04 (0.59)	0.20 (0.60)	0.18 (0.25)	−0.33 (0.38)	0.06 (0.52)	−0.20 (0.62)
		Th17	0.0003 (0.96)	0.13 (0.73)	0.0003 (0.96)	0.13 (0.73)	0.09 (0.42)	−0.22 (0.58)	0.008 (0.81)	−0.27 (0.49)
		Treg	0.33 (0.11)	0.27 (0.49)	0.33 (0.11)	0.27 (0.48)	0.17 (0.28)	−0.55 (0.12)	0.001 (0.93)	0.02 (0.97)
	CD86									
		CD28	0.18 (0.25)	−0.50 (0.17)	0.18 (0.25)	−0.50 (0.17)	0.03 (0.64)	−0.02 (0.97)	0.23 (0.19)	0.18 (0.64)
		CTLA−4	0.81 (0.001)*	0.61 (0.08)	0.81 (0.001)*	0.60 (0.08)	0.02 (0.72)	0.29 (0.46)	0.54 (0.02)*	0.54 (0.13)
		Th1	0.02 (0.69)	0.33 (0.38)	0.02 (0.69)	0.33 (0.38)	0.07 (0.48)	0.20 (0.60)	0.15 (0.31)	0.37 (0.33)
		Th2	0.33 (0.10)	0.44 (0.23)	0.33 (0.10)	0.44 (0.23)	0.09 (0.43)	−0.38 (0.32)	0.33 (0.10)	0.02 (0.97)
		Th17	0.001 (0.91)	0.30 (0.43)	0.001 (0.91)	0.30 (0.43)	0.11 (0.39)	−0.40 (0.29)	0.29 (0.13)	0.02 (0.97)
		Treg	0.005 (0.84)	0.52 (0.15)	0.005 (0.84)	0.51 (0.15)	0.04 (0.59)	−0.13 (0.73)	0.10 (0.39)	0.53 (0.14)

Treg, T regulatory lymphocytes; data are presented by *R*-squared (*R*^2^) and Spearman’s coefficient (ρ), and respective *p*-value. Bold, gray background and asterisks (*) indicates a signifficant difference (*p* < 0.05).

Analysis of the interaction between CD80 and CD86 of monocyte subsets in TRIPO cultures of NI, IND, and CARD patients revealed a significant association between CD80^+^ intermediate monocytes and CD28^+^ T cells in NI individuals (*R*^2^ = 0.84/*p* = 0.01). We observed a significant association between CD86^+^ classical monocytes and CTLA-4^+^ T cells in IND (*R*^2^ = 0.55/*p* = 0.04) and CARD (*R*^2^ = 0.81/*p* = 0.001) patients. We also found a significant association (*R*^2^ = 0.57/*p* = 0.03) and positive correlation (ρ = 0.86/*p* = 0.007) between CD80^+^ intermediate monocytes and CTLA-4^+^ T cells of IND patients. In addition, there was a negative correlation between CD80^+^ non-classical monocytes and CTLA-4^+^ T cells in CARD patients (ρ = −0.80/*p* = 0.00). A significant association between CD86^+^ non-classical monocytes and CTLA-4^+^ T cells was observed in CARD patients (*R*^2^ = 0.54/*p* = 0.02) (Table 1).

Next, we used both linear regression and correlation analyzes to explore the relationship between the expression of CD80 and CD86 by total monocytes and their subsets in driving a Th1, Th2, Th17, and Treg response in TRIPO cultures of NI, IND, and CARD individuals. A negative correlation between CD80^+^ with Treg cells was found in total monocytes of NI (ρ = −0.94/*p* = 0.00) and IND (ρ = −0.73/*p* = 0.02) patients (Table 1).

Analysis of the interaction between CD80 and CD86 in the monocyte subsets of TRIPO cultures of NI, IND, and CARD patients revealed a significant association between CD86^+^ non-classical monocytes and Treg cells of NI individuals (*R*^2^ = 0.79/*p* = 0.02). In addition, we observed a negative correlation between CD86^+^ classical monocytes (ρ = −0.78/*p* = 0.01) and CD86^+^ non-classical monocytes (ρ = −0.73/*p* = 0.02) with Treg cells of IND individuals. We also found a positive correlation between CD80 and CD86 with Th2 in classical (ρ = 0.75/*p* = 0.02) and non-classical monocytes (ρ = 0.68/*p* = 0.04) of IND patients, respectively (Table 1).

## Discussion

Monocytes are important cell of the innate immune system by their capacity to process and present antigens, produce cytokines, express co-stimulatory molecules, and activate the subsequent adaptive immune response (35). However, little is known about monocytes subsets in Chagas disease and the influence of CD80 and CD86 co-stimulatory expression in the modulation of the immune response in Chagas patients. Herein, we characterized the functional-phenotypic profile of total monocytes and subsets and investigated the effect of CD80 and CD86 expression by monocytes in the activation of Th subsets. We observed a reduced frequency of total monocytes of CARD patients, but not in IND group. In addition, a reduction in the frequency of the intermediate monocytes was also observed in CARD group. It has been proposed that the intermediate monocytes are a transitional population bridging between the classical and non-classical subsets (26). We propose that, in the presence of the *T. cruzi* antigen, these cells may differentiate from classic and/or non-classic monocytes, being important to modulate the immune response. Previously, studies have reported that monocytes could be involved in the downregulation of the immune response by the production of the anti-inflammatory cytokine IL-10 in Chagas disease (17, 32). Thus, we suggest that *T. cruzi* may decrease the frequency of monocytes of CARD patients, mainly of intermediate monocytes, thereby contributing to a pro-inflammatory environment that may be involved in the tissue damage observed in these patients.

We demonstrated that the expression of molecules associated with antigen recognition was reduced in Chagas patients, mainly the TLR-4 and TLR-9 expression in asymptomatic group. TLR-9 activation plays a primary role in MyD88 induction that is dependent on the synthesis of IL-12 and IFN-γ during *T. cruzi* experimental infection and contributes to the formation of a pro-inflammatory environment (44). The downregulation of these receptors in monocytes could be an important event in directing a reduction in phagocytic capacity of these cells as well may be a mechanism developed by asymptomatic patients to control exacerbated inflammation and downregulate the immune response. Furthermore, TLR-2 and TLR-4 expression are associated with the production of pro-inflammatory cytokines in Chagas’ cardiomyopathy, while in asymptomatic patients these receptors as predominantly associated with IL-10 and TGF-β production (41). In agreement, we observed that Chagas patients showed higher expression of IL-12, and only IND patients demonstrated increased of the IL-10 production. These results suggest that monocytes of IND and CARD patients induce an inflammatory response by producing pro-inflammatory cytokines, such as IL-12, that trigger a Th1 lymphocyte-mediate response to control the infection. However, once again, only asymptomatic patients appear to regulate the inflammatory response by the production of the anti-inflammatory cytokine IL-10, thus averting the progression of inflammation.

We also demonstrated an increase of HLA-DR expression on the classical monocytes of Chagas disease patients. In the other hand, lower expression of this antigen presentation molecule was detected in intermediate monocytes only of IND individuals. The main characteristics of classical monocytes are their high-phagocytic capacity and pro-inflammatory profile (22, 25). Our group was demonstrated that impaired phagocytic capacity driven by downregulation of HLA-DR in monocytes supports the occurrence of particularities in the APC-activation-arm in IND (12). We suggest that HLA-DR expression by classical monocytes of Chagas patients may be associated with the increase of phagocytosis ability and antigenic presentation of these cells to control the infection, as well to activate the T cell-mediated immune response. In contrast, the lower expression of HLA-DR in intermediate monocytes from asymptomatic patients may be a way of reducing inflammation and avoiding tissue damage.

CD80 and CD86 co-stimulatory molecules in monocytes from patients with Chagas disease following induction by *T. cruzi* recombinant antigens are important for the activation of T cells (43). However, no study to date has demonstrated the expression of these molecules in the different monocytes subsets in Chagas disease. We observed that Chagas patients had increased CD80 expression by classical monocytes. On the other hand, only the IND group showed high-CD86 expression by total monocytes and all subsets. Moreover, CD86 expression in these cells of asymptomatic patients was higher than CD80 expression. We propose that CD80 expression may be involved in the activation of T cells in Chagas patients, while CD86 might be associated with the inhibition of them, downmodulating the responses of effector T cells in asymptomatic patients. Previous studies have shown that exposure of monocytes to *T. cruzi* leads to an increase in the frequency of CD80 by monocytes of IND and CARD patients (43), whereas CD86 expression is decreased in CARD but not in IND patients (35). We suggest that these co-stimulatory molecules have opposing functions and probably are a key role in the T cells activation in Chagas disease.

To confirm the effect of the co-stimulatory molecules CD80 and CD86 on the activation or inhibition of T lymphocytes, we evaluated the relationship between these molecules and their ligands. CD4^+^ CTLA-4^+^ T lymphocytes were increased in Chagas patients, mainly in IND group. We also observed an association between CD80 and CD28, and between CD86 and CTLA-4. It is possible that, after *T. cruzi* infection, CD80 binds to CD28 and promotes the activation of CD4^+^ T lymphocytes and triggering an inflammatory response. On the other hand, the association of CD86 and CTLA-4 in asymptomatic patients suggests the inhibition of T cells and downmodulation of the immune response. Corroborating with our results, Souza et al. showed a higher frequency of this inhibitory molecule in IND but not in CARD patients (35). CTLA-4 also implicated in the modulation of the immune response against *T. cruzi* by affecting the mechanisms that control IFN-γ and nitric oxide production during the acute phase of Chagas disease (45). We propose that the interaction of CD86 with CTLA-4 in asymptomatic patients may be a mechanism of immunoregulation to prevent tissue damage and the pathology progression.

Infected patients demonstrated a lower frequency of Th1, Th2, and Th17 subsets than NI group. In contrast, the proportion of Treg cells was the highest in asymptomatic patients. Furthermore, only CD86 was associated with Treg cells, while CD80 showed a negative correlation with these regulatory cells only in the groups without cardiomyopathy (NI and IND). We believe that CD86 may activate Treg cells, especially in IND patients, once they shown higher CD86 expression and Treg proportion. It has been proposed that CD80 expression in APCs induces Th1 differentiation, while CD86 drives Th2 profile (46–48). Conversely, the higher frequency of Treg cells was associated with better clinical prognosis in Chagas disease patients without CARD changes (49–51). We suggest that CD86 is a key molecule to direct immunoregulation in asymptomatic patients, control immune response, and prevent progressive fibrosis in the heart.

Survival of *T. cruzi* in the host depends on it escaping from immune recognition and/or it evading immune effector mechanisms. Several studies revealed that proteins secreted by the protozoan in the bloodstream, glycosylphosphatidylinositol-mucins molecules present in the parasite surface, and the blockade of complement pathways are strategies used by *T. cruzi* to escape immune recognition and delay the progression of immune response (52–54). The capacity of the parasite to survive has been attributed to the suppression of immune responsiveness. Infection of macrophages favors the secretion of anti-inflammatory cytokines such as IL-10 and TGF-β, which impair the development of protective immune responses and help the spread of the infection (55, 56). Here, we suggest that CD86 expression may control the immune response by binding to CTLA-4 and activating Treg lymphocyte in IND, but not CARD patients. These results suggest that *T. cruzi* infection induces an increase in CD86 expression on monocytes, thus contributing to the inhibition of T cells and restrain the immune response.

The present study is the first to reveal the functional-phenotypic profile of monocytes subsets in Chagas disease. We proposed that CD86 may be involved in immunoregulation by its association with CTLA-4 in asymptomatic patients. CD86 and CTLA-4 interaction may influence Treg activation, and this could represent a new strategy to control inflammation and tissue damage. Further studies are necessary to better understand this putative evasion mechanism. This study advances the current knowledge about the immunoregulatory mechanisms mediated by CD86, CTLA-4, and by Treg cells in Chagas disease.

## Ethics Statement

This study was carried out in accordance with the recommendations of the Ethics Committee of the René Rachou Institute with written informed consent from all subjects. All subjects gave written informed consent in accordance with the Declaration of Helsinki. The protocol was approved by the Ethics Committee of the René Rachou Institute, FIOCRUZ (15/2011).

## Author Contributions

BP performed the experiments, data analysis, and wrote the paper. NM delineated the experiments, discussed the results, and made the figures. AT-C gave statistical analysis support and discussed the results. SE-S carried out the clinical assessment. DR and TF-C helped to write the manuscript and prepare the samples. RC-O and WD contributed to discussions of the results and scientific resources. JG acquired and administered funding resources, and coordinated the study. All authors contributed to manuscript revision, read, and approved the submitted version.

## Conflict of Interest Statement

The authors declare that the research was conducted in the absence of any commercial or financial relationships that could be construed as a potential conflict of interest.
